# The effectiveness of nanobiochar for reducing phytotoxicity and improving soil remediation in cadmium-contaminated soil

**DOI:** 10.1038/s41598-020-57954-3

**Published:** 2020-01-21

**Authors:** Wei Liu, Yulong Li, Ya Feng, Jianchen Qiao, Huiwei Zhao, Jixing Xie, Yanyan Fang, Shigang Shen, Shuxuan Liang

**Affiliations:** 1 College of Chemistry and Environmental Science, Hebei University, Baoding, 071002 China; 2 National Semi-Arid Agricultural Technology Research Center, Shijiazhuang, 050051 China

**Keywords:** Soil microbiology, Pollution remediation

## Abstract

There is growing concern that Cd in soils can be transferred to plants, resulting in phytotoxicity and threats to human health via the food chain. Biochar has been reported to be a soil amendment capable of reducing the bioavailability of metals in soil by electrostatic interactions, ionic exchange and the specific binding of metal ions by surface ligands. To determine the effects of Cd contamination and nanobiochar on the growth characteristics of plants, the dynamics of Cd in soil were explored in Petri dish and pot experiments (0%, 0.2%, 0.5% and 1% nanobiochar), respectively. The diversity, distribution and composition of the bacterial community in treated soil were monitored by high-throughput sequencing. The results showed that the germination potential and height and weight of plants were significantly decreased in Cd-treated soil samples (*P* < 0.05). The Cd content of *Brassica chinensis* L. in the treated soil groups was lower than that in the untreated soil groups (*P* < 0.05) after nanobiochar application. The application of biochar significantly improved the microbial biomass, microorganism abundance and diversity of *Actinobacteria* and *Bacteroidetes* in Cd-contaminated soil and reduced the diversity of *Proteobacteria*, which was relatively more persistent than in the contaminated sites without biochar application. The results of this study provide theoretical and technical support for understanding the environmental behavior of nanopassivators, thus enhancing the role of biochar in the remediation of soil pollution.

## Introduction

Cd pollution often occurs in soils due to numerous human activities, such as mining, sludge and sewage application, irrigation, chemical fertilization and pesticide application^1^. In China, Cd pollution has worsened. It was reported by the Ministry of Environmental Protection, P. R. China, that as of 2014, 7% of all cultivated areas contained Cd-contaminated soil. There is growing concern regarding Cd contamination because excess Cd exposure can directly cause both noncarcinogenic and carcinogenic risks^2^. Cd in soils can be readily taken up by plant roots and transferred to aboveground plant parts, resulting in phytotoxicity, which will indirectly cause a threat to animal and human health through the food chain^2–5^. A survey showed that approximately 59% of vegetables in Taiyuan, Shanxi Province, China had excessive Cd concentrations^6^. The medium and high comprehensive potential ecological risk point was found in 57.15% of vegetables in the Shijiazhuang area, Hebei Province, China^7^. In addition, excessive concentrations of Cd in soil can affect soil microorganisms and their activities^8^. Previous reports also showed a decrease in soil microbial biomass^9^ and changes in the soil microbial community structure as a result of excessive Cd in the soil^10^. Therefore, it is important to find an effective method for the remediation of Cd-contaminated soil.

Soil amendments are used to reduce the bioavailability of metals in soils by immobilizing the metals into stable forms^11^. In recent years, many types of environmentally friendly soil amendments, such as biochar, have been reported and have gained much attention. Biochar is produced through incomplete pyrolysis of biomass, such as wood waste, and organic materials, such as agricultural crop residues, under limited oxygen concentrations^12^.. Previous reports from different regions have demonstrated the effectiveness of biochar in heavy metal reduction due to its sorptive characteristics^13–15^. The addition of biochar may modify soil chemical properties, providing suitable conditions for heavy metal immobilization and subsequently reducing heavy metal uptake by plants^16^. For example, in Egypt, Wabel and his colleagues observed that the addition of *Conocarpus erectus* L. biochar significantly reduced shoot heavy metal concentrations in maize plants^17^. Though these potential benefits have been reported, decreases in crop productivity have also been reported with specific combinations of soil and biochar^18,19^. Therefore, the negative implications for plants and soil animals associated with biochar technology need to be better understood^20^.

In recent years, nanoremediation has played an increasingly important role in environmental improvement and pollution prevention, detection, monitoring and remediation, including pollution from chlorinated compounds, hydrocarbons, organic compounds and heavy metals^21–23^. These nanoscale materials play a role in heavy metals absorption due to their large microinterface, micropores and high surface area. Therefore, nanomaterials with potential to exert beneficial effects on the environment are of great interest.

Soil is considered to have multiple components, forming an open biochemical system. Microorganisms play an important role in monitoring ecosystem health and function, and any changes in the microbial community will affect the whole ecosystem^24^. A better understanding of how nanobiochar influences the community composition and activity of microorganisms in Cd contaminated soil can aid in the evaluation of the remediation effects of amendments in heavy metal polluted soil. Therefore, we hypothesize that the *in situ* nanomaterial remediation technology in combination with biochar remediation technology could play an increasingly important role in promoting plant growth and enhancing soil properties. Thus, we assessed the inhibition of plant growth by Cd and the effectiveness of nanobiochar for enhancing plant growth and soil microbial diversity under excess Cd. A lab experiment was conducted using different concentrations of nanobiochar and Cd^2+^ solution to assess the phytotoxic effects of Cd^2+^ on the germination of plants and the restoration capability of nanobiochar. In addition, a pot experiment was carried out in soil with simulated cadmium-contamination to better understand the effects of nanobiochar on the dynamics of the cadmium content and the microbial community in contaminated soil.

## Materials and Methods

### Soil collection

Cultivated soil was collected from the Baoding suburb, Hebei Province, China. Air-dried soil was sieved through a 2 mm screen and then mixed with Cd(NO_3_)_2_ powder to reach a Cd concentration of 10 mg kg^−1^. The actual measurement of the Cd content in the soil sample was 9.64 mg kg^−1^. Soil characteristics (e.g., pH, organic carbon, total nitrogen, total phosphorus, cation exchange capacity, and Cd content) were determined by using the methods of Bowman and Hutka^25^ (Table 1).

**Table 1:** Soil sample properties.

Soil type	pH (1:5)	Organic matter (mg/kg)	Total nitrogen (g/kg)	Total phosphorus (mg/kg)	CEC (cmol/kg)	Cd content (mg/kg)
Cinnamon soil	8.5 ± 0.12	27.18 ± 0.65	4.74 ± 0.15	9.76 ± 0.31	156.04 ± 5.73	9.64 ± 0.21

CEC: cation exchange capacity.

The biochar used for the pot cultivation experiments was from wheat straw that had undergone pyrolysis at 350–550 °C in a commercial pyrolysis reactor at the Sanli New Energy Company, China. The main characteristics of the biochar were as follows: pH (H_2_O), 8.23; specific surface area, 1331 m^2^/g; organic matter, 125 g/kg; potassium, 55.46 g/kg; phosphorus, 4.5 g/kg; methylene blue (MB) adsorption capacity, 8.3 mg/g; and iodine adsorption capacity, 100 mg/g^26^.

### Germination test

*Preparation of particle suspensions and cadmium solution*. The nanoparticles were suspended directly in double distilled water (DDW) and dispersed using a mechanical stirrer for 30 min. Small magnetic bars were placed in the suspensions for stirring before use to avoid aggregation of the particles.

*Sprouting experiment*. Seeds of four vegetables (tomato, carrot, lettuce, and cucumber) were selected. Fifteen seeds of each vegetable were placed in Petri dishes and incubated in the dark at 25 ± 2 °C. Observations were recorded after 48 h of treatment with different concentrations of nanobiochar and cadmium chloride solution. A seed was considered germinated when its plumule was ≥ 2 mm. Controls were obtained by moistening the filter papers with 5 mL of deionized water.

*Treatments*. In the single toxicant tests, the concentrations of Cd^2+^ were 0, 50, 80, 100, 120 and 150 mg L^−1,^ and the concentrations of nanobiochar were 0, 50, 100, 200, 500 mg L^−1^. In the combined tests, the cadmium concentration was maintained at 8 mg/L(EC_50_) for all treatments, and then a series of biochar concentrations of 0, 50, 100, 150, 200, 300 and 500 mg/L were added to determine the concentration with the best effects.

#### Plant germination

The germination potential and germination percentage were calculated for 7 d and 10 d using the following equation^27^:

$$Germination\,percentage=\frac{TNG}{TNP}\ast 100 \% $$

where TNG is the total number of seeds germinated at the peak of germination and TNP is the total number of seeds planted.

### Pot experiment

The pot experiment was performed at Hebei University, China. Nanobiochar was added at 0%, 0.2%, 0.5%, and 1% to Cd-contaminated soil samples at a temperature of 18–25 °C and relative humidity of 60–70%. Deionized water was added to maintain the soil water holding capacity at 60% for 30 d for the pot experiment.

Chemical fertilizer was applied to the four soil samples at an N:P:K ratio of 1.5:1:1.5. Next, the soil samples (2500 g each) were placed into plastic pots and incubated for 10 d.

Ten *Brassica chinensis* L. seeds (purchased from the subsidiary corporation of The Dutch Carter Biological Technology Company, Shouguang, China) were sown into each pot, and after 7 d, thinning was performed, leaving 5 seedlings per plot. Five replicates of each plant were employed. The plants were irrigated to compensate for evaporation loss. All treatments were randomly arranged and subjected to the same growth conditions. During the growth period, fertilization was applied to avoid stress according to the growth conditions of the plants.

#### Soil and plant sampling

*Brassica chinensis* L. samples were collected 0, 15, 30, 45, 60 and 90 d after planting. Duplicate composite samples were taken from each pot at each sampling time. Plants and their associated root material were removed from the pots with a trowel. At the same time, the soil adhering to the roots (100 g) was also collected and used as the rhizosphere soil sample.

When sampling, plants in each pot were placed in a plastic bag and combined to form one sample. Samples were immediately transported in a cooled box to the laboratory and processed in less than 6 h after removal from the pot. The roots were shaken vigorously to separate the soil that was not tightly adhering to the roots. Root-attached soil was then squeezed from the roots with a gloved hand and mixed evenly. Soil subsamples for DNA extraction were taken and stored at −80 °C until analyses were performed. Air-dried soil samples were sieved to < 0.25 mm and were used for measurement of the cadmium content^28^.

#### Chemical analysis

The *Brassica chinensis* L. samples were washed with deionized water and measured for plant height and fresh weight. Dry weight was measured after the samples were oven-dried at 105 °C for 30 min and then dried at 60 °C until no change in weight was observed.

For heavy metal analysis, the plant tissues were ground and passed through a 0.15 mm sieve. Then, the plant materials (0.5 g) were digested with HNO_3_ (6 mL), HF (2 mL) and H_2_O_2_ (2 mL) for 12 h, and the remaining plant tissues were filtered through a 0.22-μm PTF filter and analyzed by Inductively Coupled Plasma Optical Emission Spectrometry (ICP-MS)(Agilent 7500a, Agilent, America)^29^. Similarly, the Cd concentration in soil samples was determined using microwave digestion with concentrated HNO_3_, HF, H_2_O_2_ and measured by ICP-MS as well.

#### Determination of the speciation of soil Cd

The determination of the speciation of soil Cd was performed by a modified sequential extraction procedure^29,30^. Five Cd species, including exchangeable, bound to carbonates, bound to Fe and Mn oxides, bound to organic matter, and residual, were determined. Briefly, soil samples (1.0 g) were weighed into 50 ml polyethylene centrifuge tubes. The sequential extraction process involved five stages as follows:

1. Incubation in 8 ml 1 moL/L MgCl_2_ (pH = 7.0) at 25 °C for 1 h;
2. Incubation in 8 ml 1 moL/L CH_3_COONa (pH = 5.0) at 25 °C for 5 h;
3. Incubation in 20 ml 0.04 moL/L NH_2_OH·HCl and 25% CH_3_COOH (pH = 2.0) at 95 °C for 6 h;
4. Incubation in 3 ml 0.02 moL/L HNO_3_ and 5 ml 30% H_2_O_2_ at 85 °C for 2 h, then 5 ml 30% H_2_O_2_ at 95 °C for 6 h. After cooling to room temperature, further incubation in 3.2 M NH_4_OAc in 20% (v/v) HNO_3_ (5 mL) for 0.5 h with continuous agitation;
5. Incubation in an acidic mixture of HF-HClO_4_-HNO_3_-HCl with digestion.

Following each extraction stage, the solutions were centrifuged for 10 min at 4000 rpm, and the supernatant was filtered through a 0.45 µm filter. The soils were washed with deionized water and centrifuged prior to the next extraction stage, and the washings were discarded. The Cd contents in the filtered supernatants were measured by atomic absorption spectrophotometer.

### Microbial analyses

#### DNA isolation and polymerase chain reaction (PCR)

The DNA of the total microbial community was extracted from 0.5 g samples using a bead beating method (FastDNA SPIN Kit for Soil, Bio101 Inc., USA) according to the manufacturer's instructions. Then, the V3 region of 16 S rDNA was amplified with the primers 357 F GC clamp (5′-CGCCCGCCGCGCCCCGCGCCCGGCCCGCCGCCCCCGCCCCCCTACGGGAGGCAGCAG-3′) and 518 R (5′-ATTACCGCGGCTGCTGG-3′). PCR was performed using a Hybrid PCR Express thermal cycler (Bio-Rad, USA) in a 50 μl volume containing 10 ng of DNA template, 25 pM of each primer, 2.5 Mm deoxynucleotide triphosphates (dNTPs, Promega, USA), PCR buffer (Applied Biosystems, USA), 0.1 mM MgCl_2_ solution (Sigma), and 1 U of Taq polymerase (Applied Biosystems, USA). Negative controls did not contain template DNA. The PCR conditions for amplification were as follows: initial denaturation at 94 °C for 3 min; 34 cycles of denaturation at 94 °C for 50 s, annealing at 56 °C for 1 min and DNA extension at 72 °C for 30 s; and a final extension step at 72 °C for 10 min. Amplified DNA was detected on a 1% agarose gel stained with SYBRTM Green I (Sigma, USA) and visualized by a Fluor-S MultiImager (Bio-Rad, USA).

High-throughput sequencing was conducted by Personalbio (Personal Biotechnology Co., Ltd, Shanghai, China).

#### Biodiversity and phylogenetic analyses

The summary single command in Mothur software was used to evaluate the biodiversity of 4 common species according to the OTU data. A Venn diagram was also constructed using Mothur based on the clustering analysis.

### Statistical analysis

Data were expressed as the average ± standard deviation (SD). Statistical analysis was performed using Microsoft Excel and SPSS 12.0. Significance was set at *P* < 0.05. Significant differences in germination were examined using a t-test with a 95% confidence interval.

## Results

### Effects of nanobiochar on seed germination and plant growth

#### Effects of nanobiochar on seed germination

As the concentration of cadmium in the solution increased, the germination percentage of cucumber, carrot and tomato increased and then decreased, and the maximum germination was observed with the 20 mg/L cadmium chloride solution. However, the percentage of lettuce germination decreased with increasing Cd concentration. These results indicated that the germination of cucumber, tomato, lettuce and carrot tended to decrease as the Cd concentration increased.

**Figure 1:**
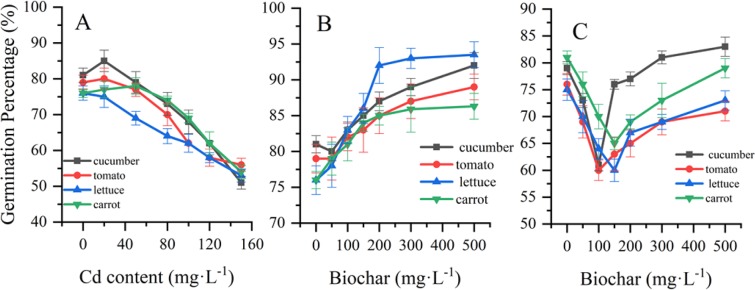
The effects of biochar on the germination of plant seeds after 48 h of exposure to Cd-contaminated solution (**A**) cadmium only treatment; (**B**) nanobiochar only treatment; (**C**) cadmium and nanobiochar cotreatment.

To detect the effect of nanobiochar on germination, the four vegetable seeds were exposed to different concentrations of nanobiochar suspension (Fig. 1B). The results showed that the germination percentages increased by 11%, 10%, 17.5% and 10.3%, respectively, when grown on 500 mg/l nanobiochar-treated filter paper compared to the control (all *P* < 0.05). Although there was a slight decrease in the germination percentage for cucumber and tomato in the 50 mg/l nanobiochar-treated group, an increasing tendency was found in the nanobiochar suspension treatment (Fig. 1B).

When treated with Cd and nanobiochar suspension together, the germination rates initially decreased and then increased with higher concentrations of biochar (Fig. 1C). These data suggest that nanobiochar can ameliorate the decreased germination caused by Cd contamination.

#### Plant growth and metal uptake

**Figure 2:**
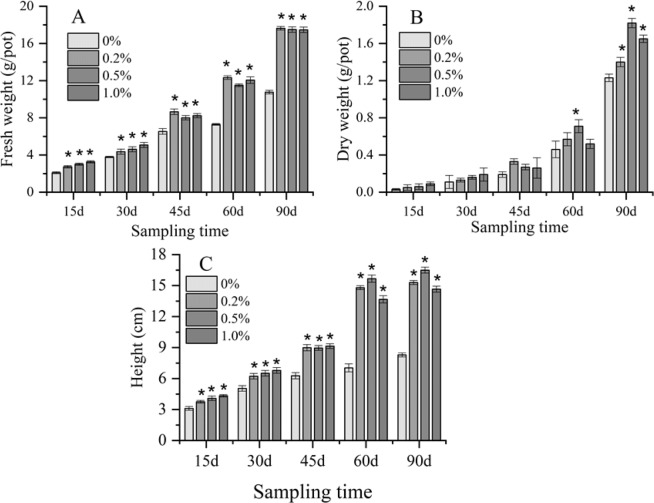
The effects of nanobiochar on the weight and height of *Brassica chinensis* L. in Cd-contaminated soil (**A**) fresh weight; (**B**) dry weight; (**C**) height. *Indicates significant differences between the treated group and the control group at *P* < 0.05.

To evaluate the effects of nanobiochar on plant growth, *Brassica chinensis* L. was collected for measurements at 15 d, 30 d, 45 d, 60 d and 90 d after planting. The results showed that fresh weight (Fig. 2A) and height of *Brassica chinensis* L. (Fig. 2C) were significantly increased under nanobiochar application to the Cd-contaminated soil (all *P* < 0.05). High concentrations of nanobiochar did not significantly improve the dry weight (Fig. 2B) at the start of the experiment (before 45 d), but the dry weight increased markedly at 60 d under the 0.5% nanobiochar treatment (*P* < 0.05). Additionally, the dry weight of *Brassica chinensis* L. was much higher in the nanobiochar treatment groups at 90 d than that of the control group (*P* < 0.05).

**Figure 3:**
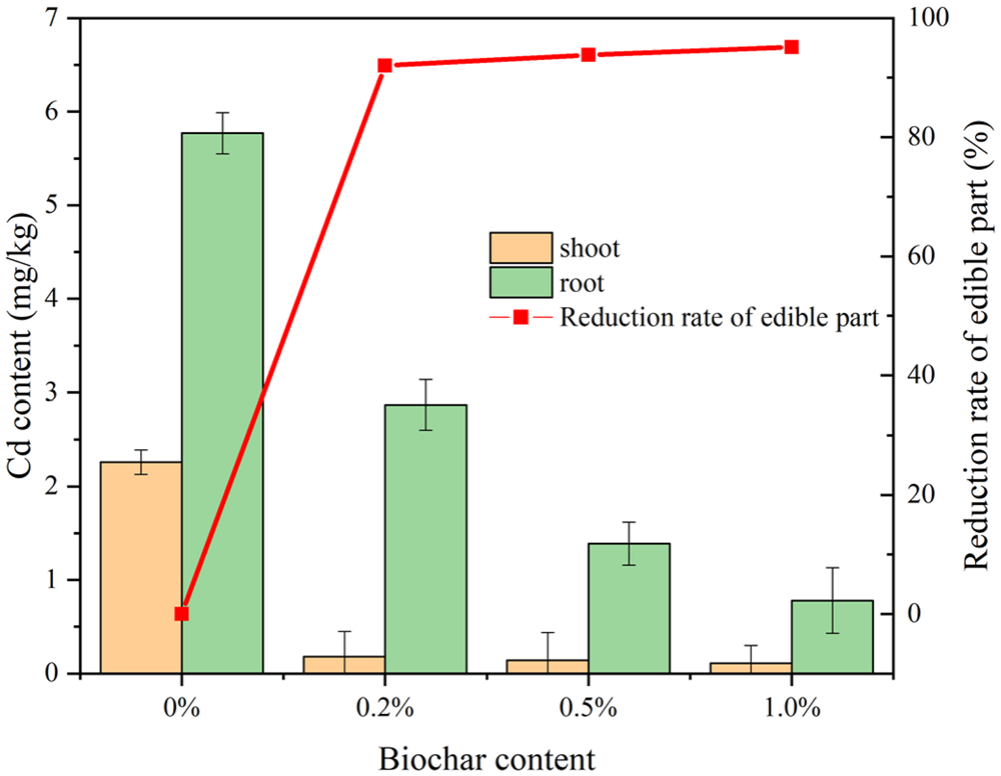
Cd content in *Brassica chinensis* L.

The *Brassica chinensis* L. in the treated soil groups showed lower Cd contents relative to the untreated soil group (Fig. 3). In the untreated soil group, the Cd content in the root (5.77 mg kg^−1^) was much higher than that in the aboveground parts (2.26 mg kg^−1^) (*P* < 0.05). The Cd content in both the root and aboveground parts of *Brassica chinensis* L. significantly decreased by 95.1% and 86.5%, respectively, in the 1% nanobiochar-treated soil group compared with the untreated groups (all *P* < 0.05).

####  Cd content in soil

**Figure 4:**
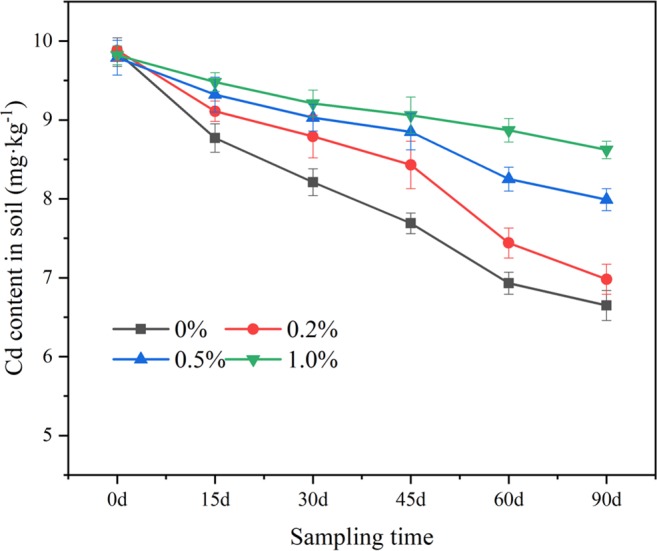
Cd content in soil groups.

The Cd content in the soil is presented in Fig. 4. The Cd content increased with the increase in nanobiochar in the soil, although the Cd content in the soil decreased as the plant grew because of plant uptake and accumulation after planting.

**Figure 5:**
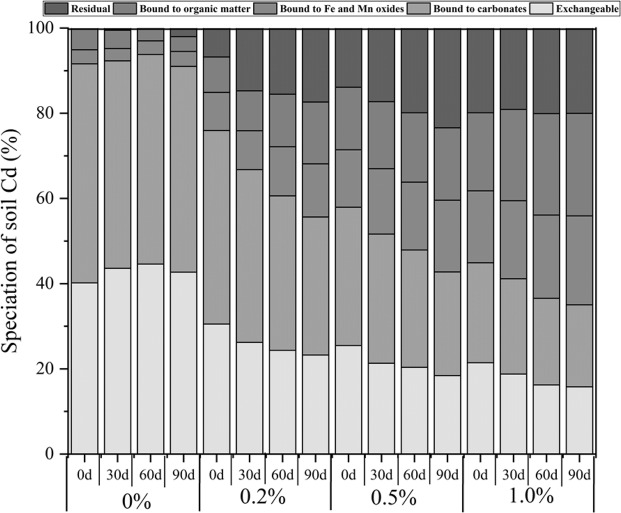
Speciation of soil Cd.

Quantitative speciation of Cd in the treated and untreated soils was performed (Fig. 5). In general, the results indicated that in the untreated soil sample, Cd was mainly composed of the exchangeable and bound to carbonate fractions, followed by the residual fraction. The two fractions accounted for more than 40.2%, 48.3%, and 90.5% of the total concentrations in soils. A higher percentage of Fe-Mn oxide, organic and residual fractions were found in the soils with nanobiochar application. Furthermore, the Fe-Mn oxide, organic and residual ratios increased with an increased amount of nanobiochar.

### Diversity of soil microorganisms

The microbial biodiversity in the different variants was analyzed in terms of the abundance-based coverage estimator (ACE)^31^, Chao1^31^ and Shannon indices^32^, as shown in Table 2. The soil groups treated with 0.5% and 1% nanobiochar showed higher species richness than the soil groups treated with 0% and 0.2% nanobiochar (Table 2).

**Table 2:** Soil species richness estimation.

Sample	Chao1	ACE	Shannon
0%	1202.7	1169.4	4.60
0.2%	983.8	1027.6	5.18
0.5%	1559.0	1576.1	5.95
1%	1270.9	1166.4	6.31

Note: 0%, 0.2%, 0.5%, 1.0% indicates the ratio of nanobiochar to soil (W/W); Chao1, ACE, Shannon: microbial community diversity index.

**Figure 6:**
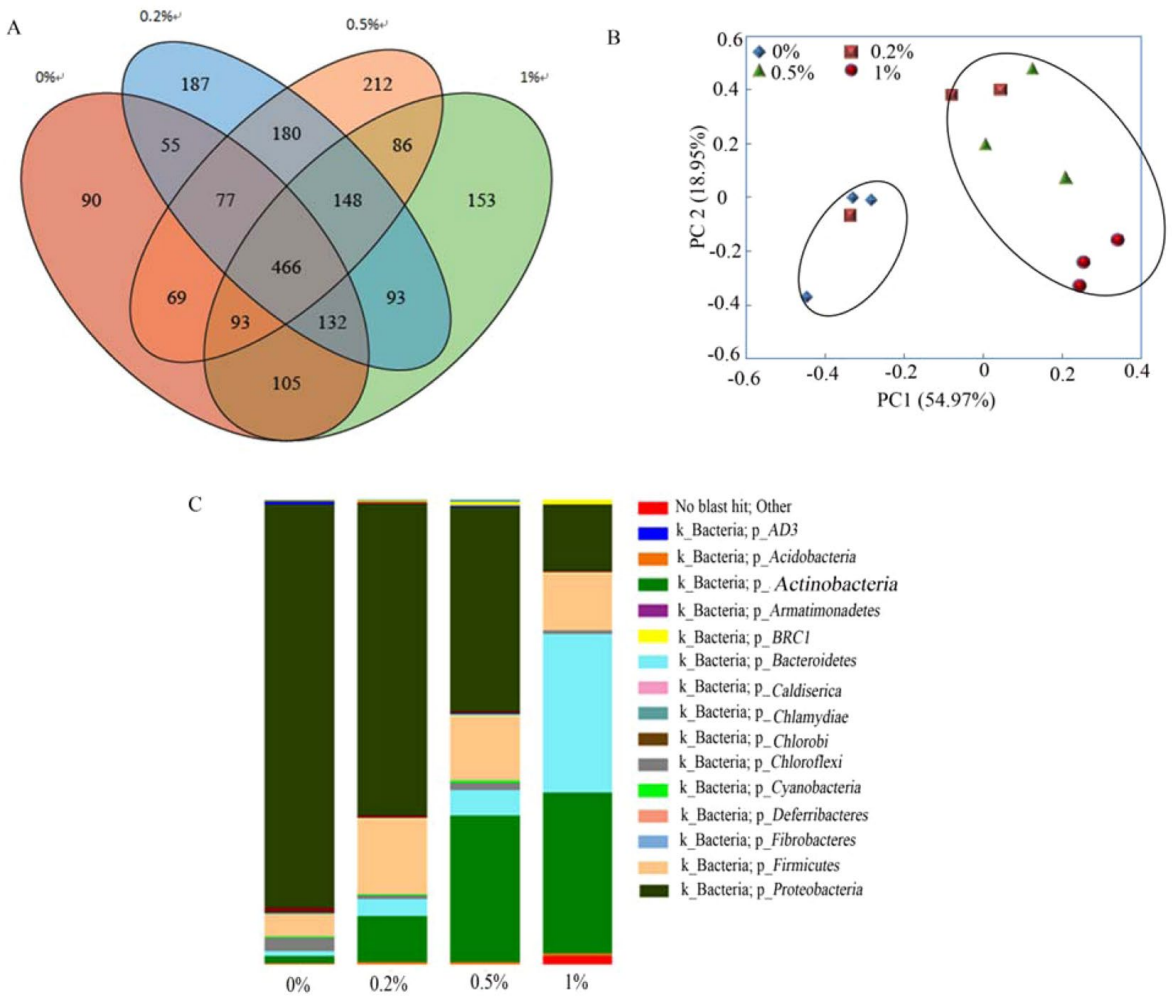
Biodiversity of soil organisms. (**A**) Venn diagram showing the microbial biomass in different soil samples; (**B**) Principal component analysis (PCA) in different treated soil samples; (**C**) Relative abundance of dominant species.

Nanobiochar-treated soil samples showed more microbial biomass than nontreated soil samples (Fig. 6A). Comparison of microorganism abundance showed a different relative abundance pattern in each soil sample (Fig. 6C). The dominant microorganism phylum was *Proteobacteria* for soil samples treated by 0% and 0.2% nanobiochar. The 0.5% nanobiochar-treated soil sample was predominantly inhabited by *Proteobacteria* and *Actinobacteria*, while the 1% nanobiochar-treated soil sample was predominantly inhabited by *Actinobacteria* and *Bacteroidetes*.

Principal component analysis (PCA) showed that the soil microbial community could be identified by PC1 and PC2. The soil microbial communities in 0.5% and 1% nanobiochar-treated soil samples were positively correlated with PC1, while the microbial community in the 0% treated soil group was negatively correlated with PC1. The results were consistent with the diversity of microorganisms, as shown in Fig. 6, suggesting that the 0.5% and 1% nanobiochar soil treatments significantly changed the microbial community.

**Figure 7:**
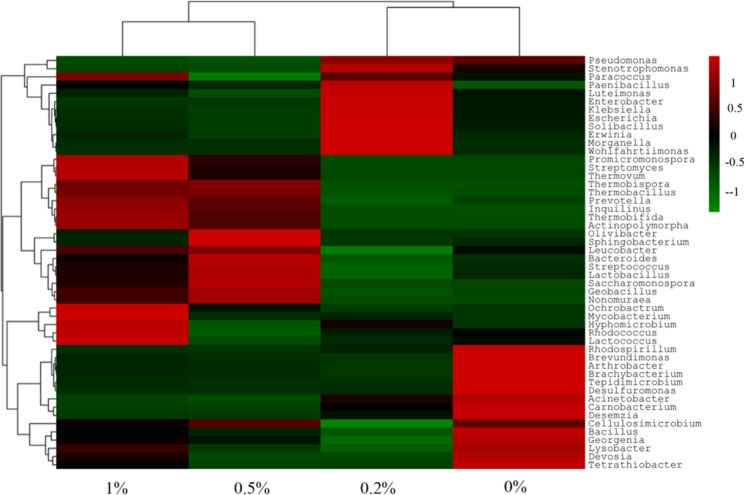
Heat map of soil microorganisms.

A heatmap was used to cluster the microorganisms at the genus level (Fig. 7). Soil bacteria were classified into 5 groups. Bacterial abundance was different among the four soil samples: in the 0% nanobiochar-treated soil sample, more bacteria were found in Group 5, including *Rhodospirillum*, *Brevundimonas*, *Arthrobacter*, *Tepidimicrobium*, etc. In the 0.2% nanobiochar-treated soil sample, Group 1 bacteria were most prominent, including *Luteimonas*, *Enterobacter*, *Klebsiella*, *Solibacillus*, etc. Group 3 bacteria were mainly distributed in the 0.5% nanobiochar-treated soil samples (such as *Olivibacter*, *Sphingobacterium*, *Lactobacillus*, *Geobacillus*, etc.), Group 2 and Group 3 bacterial species were primarily distributed in the 1% nanobiochar-treated soil sample. This indicates that bacterial abundance was associated with the extent of cadmium bioavailability.

## Discussion

The present study showed that the application of nanobiochar significantly decreased the exchangeable cadmium and improved plant growth by increasing the seed germination and the height and weight of plants. When Cd^2+^ enters the cell, it replaces the essential elements (Zn, Ca, Fe) and releases free ions, further causing oxidative damage by the Fenton reaction^33^. Therefore, the germination percentage increased significantly as the cadmium solution decreased. We then examined the Cd^2+^ content in *Brassica chinensis* L., which was planted in a soil with simulated Cd contamination, and confirmed that nanobiochar significantly reduced the Cd^2+^ content either in the roots or aboveground plant parts. A similar result was obtained by Bian *et al*.^3^. On the one hand, nanobiochar reduced the content of available cadmium in soil, reduced the uptake of cadmium by *Brassica chinensis* L., and thenreduced the phytotoxicity of cadmium. On the other hand, the potash and phosphate fertilizers in the nanobiochar provided nutrients and promoted the growth of *Brassica chinensis* L. In this study, nanobiochar increased the germination percentage of seeds in solution and promoted the growth of *Brassica chinensis* L. in cadmium-contaminated soil. After the harvest of plants, the residual cadmium content in the soil increased with the increasing biochar content, which also proved that nanobiochar reduced the uptake of cadmium in *Brassica chinensis* L. Moreover, the soil microbial diversity also indicated that the application of nanobiochar significantly increased microorganism biodiversity, which is beneficial for soil fertility. These data suggest that nanobiochar could limit the phytotoxicity caused by Cd and increase soil fertility.

The mobility and toxicity of metals are mainly dependent on metal speciation in the environmental medium. The chemical speciation of Cd in soil samples was analyzed using a modified sequential chemical extraction procedure to provide further information on metal distribution with different operationally defined geochemical phases. The metal fractionation analysis showed that the major speciations of Cd were exchangeable and bound to carbonates, which was consistent with the results reported by Wang *et al*.^29^. However, the nanobiochar application depressed the Cd amount in the exchangeable and carbonate bound pools, while it increased the Cd amount in the other three pools because of its larger specific surface area, smaller particle size and higher reaction activity through surface complexation and adsorption. The Cd in soil became unabsorbable for plants after nanobiochar application. In addition, the core-shell structures of the nanomaterial are also beneficial for available heavy metal reduction by transformation^34^. Because the metallic core acts as an electron source, the oxide shell is involved in absorbing the contaminants by surface complexation and electrostatic actions. Therefore, the combined effect of biochar and nanomaterial contributes to the decrease in the inhibition and toxicity of heavy metals in soil.

Soil organisms are suggested to play an important part in the process of biochar remediation^18^. In this study, the results showed that the application of nanobiochar significantly increased the microbial biomass because it combined the advantages of biochar with nanomaterials and provided a suitable environment for microbial growth^35^. In addition, a previous study has proved that labeled N found in plants was from biochar^36^, indicating that nanobiochar can supply a food source for soil microorganisms. Furthermore, the application of biochar provides a suitable soil environment for microorganisms by increasing porosity, soil tensile strength and water holding capacity, which in turn improves soil activity^18^.

The diversity of soil organisms was also associated with the soil environment. In Cd-contaminated soil samples, *Proteobacteria* was the dominant taxon in our study and comprised the vast majority of all organisms. Furthermore, the abundance of the dominant phylum, *Proteobacteria*, which represented approximately 80% of all classified sequences in the 0% nanobiochar treatment soil, corresponded roughly to those found in the studies of Singleton *et al*.^37^, and Uhlik *et al*.^38^. Thus, despite the different surveying efforts and sampling sites used in different studies, a variety of contaminated soils were found to contain the same dominant bacterial groups^37,38^. The dominance of these groups may be explained by the high tolerance of *Proteobacteria* to Cd^2+,^^39^. After restoration by nanobiochar, both *Actinobacteria* and *Bacteroidetes* were found in soil samples. This indicated that both soilborne *Actinobacteria* and *Bacteroidetes* may be capable of subsisting on nanobiochar as a sole C source^40,41^. These microorganisms adhere to the surface of the nanobiochar and gradually form communities and biofilms that adhere the nanobiochar to the surface of soil minerals. Therefore, microbial composition may play a key role in fixing nanobiochar carbon in soil.

Soil properties play an important role in microorganism communities. In our study, bacterial abundance was also found to be associated with the extent of soil contamination. For example, researchers found that heavy metal ions show toxicity to microorganisms by binding with the cell wall^8^. Biochar application could improve soil quality by increasing the soil organic content and provide suitable microsites for soil microorganisms to survive^42^. Our results also proved that the exchangeable form of cadmium decreased and microorganism diversity increased after the addition of nanobiochar. Therefore, the application of nanobiochar significantly reduces microorganism inhibition by promoting the transformation of Cd from exchangeable to other forms. On the other hand, nanobiochar application improves soil properties, increasing the suitability of the soil for microbial growth.

## Conclusion

The application of nanobiochar to Cd-contaminated soils was investigated by monitoring the Cd phytotoxicity, availability of Cd in soils and microbial community abundances. The results demonstrated that the application of nano-biochar significantly increases seed germination, plant biomass, and the diversity of microorganisms in Cd-contaminated soil. The application of nanobiochar can significantly reduce the available Cd in soil and positively affect the stability of cadmium ions in soil. The addition of nanobiochar also significantly improved the microbial biomass, microorganism abundance and diversity of *Actinobacteria* and *Bacteroidetes*, which are beneficial for the remediation of contaminated soil. As a low-cost material, nanobiochar is expected to be widely used in high-concentration cadmium-contaminated farmland to ensure food safety and human health. However, to meet long-term agricultural needs and realize a more practical application of this data, more extensive studies and long-term field trials are required to confirm the reliability of these findings.
